# Analysis of MDR genes expression and cross-resistance in eight drug resistant ovarian cancer cell lines

**DOI:** 10.1186/s13048-016-0278-z

**Published:** 2016-10-18

**Authors:** Radosław Januchowski, Karolina Sterzyńska, Katarzyna Zaorska, Patrycja Sosińska, Andrzej Klejewski, Maciej Brązert, Michał Nowicki, Maciej Zabel

**Affiliations:** 1 Department of Histology and Embryology, Poznan University of Medical Sciences, Swiecickiego 6 St., 61-781 Poznan, Poland; 2 Department of Pathophysiology, Poznan University of Medical Sciences, Poznan, Poland; 3 Department of Nursing, Poznan University of Medical Sciences, Poznan, Poland; 4 Division of Infertility and Reproductive Endocrinology, Department of Gynecology, Obstetrics and Gynecological Oncology, Poznan University of Medical Sciences, Poznan, Poland; 5 Department of Histology and Embryology, Wrocław Medical University, Wroclaw, Poland

**Keywords:** Drug resistance, Ovarian cancer, Chemotherapy, Drug transporters

## Abstract

**Background:**

Multiple drug resistance (MDR) of cancer cells is the main reason of intrinsic or acquired insensitivity to chemotherapy in many cancers. In this study we used ovarian cancer model of acquired drug resistance to study development of MDR.

We have developed eight drug resistant cell lines from A2780 ovarian cancer cell line: two cell lines resistant to each drug commonly used in ovarian cancer chemotherapy: cisplatin (CIS), paclitaxel (PAC), doxorubicin (DOX) and topotecan (TOP). A chemosensitivity assay - MTT was performed to assess drug cross-resistance. Quantitative real-time polymerase chain reaction and immunofluorescence were also performed to determine mRNA and protein expression of genes/proteins involved in drug resistance (P-gp, BCRP, MRP1, MRP2, MVP). Flow cytometry was used to determine the activity of drug transporters.

**Results:**

We could observe cross-resistance between PAC- and DOX-resistant cell lines. Additionally, both PAC-resistant cell lines were cross-resistant to TOP and both TOP-resistant cell lines were cross-resistant to DOX. We observed two different mechanisms of resistance to TOP related to P-gp and BCRP expression and activity. P-gp and BCRP were also involved in DOX resistance. Expression of MRP2 was increased in CIS-resistant cell lines and increased MVP expression was observed in CIS-, PAC- and TOP-, but not in DOX-resistant cell lines.

**Conclusions:**

Effectiveness of TOP and DOX in second line of chemotherapy in ovarian cancer can be limited because of their cross-resistance to PAC. Moreover, cross-resistance of PAC-resistant cell line to CIS suggests that such interaction between those drugs might also be probable in clinic.

## Background

One of the main reasons of low effectiveness of chemotherapy in cancer patients is drug resistance, which is inherent or, more often, acquired during treatment [1]. In most cases drug resistance has features of Multiple Drug Resistance (MDR). MDR is designated as an insensitivity of cancer cells not only to previously used drug but also to many other drugs with different chemical structure and mechanism of action [2]. Majority of drugs used in chemotherapy act as a cytotoxic agents then as cytostatic ones. Although cancer cells develop various mechanisms of resistance to cytotoxic drugs the first players implicated in MDR are drug transporters from ABC family. These proteins use energy from ATP hydrolysis for active removing drugs from the cancer cells [3]. The most important drug transporter is glycoprotein P (P-gp) encoded by the multidrug resistance protein 1 gene (*MDR1*, *ABCB1*) [4]. Expression of this protein was noted in over 50 % of cancers with MDR phenotype and it can be inherent or induced by chemotherapy [5]. Approximately 20 different cytotoxic drugs are substrates to P-gp [6] and two of them - paclitaxel [7] and doxorubicin [8] - are commonly used in chemotherapy of many cancers. The second most important drug transporter is breast cancer resistant protein (BCRP) encoded by *ABCG2* gene, cloned for the first time from breast cancer cell line MCF-7 [9]. The upregulated expression of BCRP was noted in many cancers including breast [10] and ovarian [11] and is known to protect cancer cells against mitoxantrone [10, 12] and topotecan [11, 12]. Other important ABC transporters implicated in MDR of cancers include MRP1 and MRP2 (MDR1-related protein 1 and MDR-related protein 2) encoded by *ABCC1* and *ABCC2* genes, respectively [3, 13, 14]. Substrates used by MRP1 are similar to those for P-gp with the exception of taxanes [6]. Among many MRP2 substrates the most important is cisplatin (CIS) and it is the most frequently used antitumor agent in cancer therapy [6, 12].

Another protein involved in MDR, but not belonging to ABC drug transporters family, is MVP/LRP major vault protein/lung resistance - related protein [15]. The upregulation of MVP/LRP expression was noted in lung cancer and was correlated with poor response to chemotherapy [16]. LRP expression increased after exposure to CIS in non-small-cell lung cancer cells [17].

To better understand the mechanisms of drug resistance development and cross-resistance to different cytotoxic drugs we used the ovarian cancer model, the most lethal gynaecological cancer [18]. Ovarian cancer seems to be an appropriate model to study mechanism of drug resistance development because it is one of the most treatable cancers at the beginning of the therapy [18]. Unfortunately, most of the patients with good response to chemotherapy have recurrence with acquired MDR [18, 19]. As a result, the second line of chemotherapy is not curative [18].

The current research that improves the knowledge about drug resistance development is based mainly on drug sensitive and resistant cancer cell lines. However, most studies are limited to only one or two resistant cell lines. Therefore, we have developed eight drug resistant cell lines from one parental A2780 ovarian cancer cell line to make model of drug resistance more accurate and effective. Cell lines used in our experiments were resistant to cytotoxic drugs from the first line chemotherapy regimen of ovarian cancer - paclitaxel (PAC) and cisplatin (CIS) [20] - as well as to two drugs commonly used in the second line of chemotherapy - doxorubicin (DOX) and topotecan (TOP) [21, 22]. Such model enable us the comparison not only between the development of drug resistance for drugs of the first and the second line of chemotherapy, but also let us observe differences in twin cell lines resistant to the same cytotoxic drug.

Our study had four main goals: 1. To compare the mechanism of drug resistance to cytotoxic agents used in the first and the second line of ovarian cancer chemotherapy. 2. To determine the expression of the main genes in drug resistant cell lines. 3. To compare the cross-resistance between cell lines resistant to investigated drugs. 4. To determine the differences and similarities between twin cell lines resistant to the same cytotoxic drug.

## Methods

### Reagents and antibodies

CIS, DOX, TOP, and PAC were obtained from Sigma (St. Louis, MO). RPMI-1640 medium, fetal bovine serum, antibiotic-antimycotic solution, and L-glutamine were also purchased from Sigma (St. Louis, MO). A Cell Proliferation Kit I (MTT) was purchased from Roche Diagnostics GmbH (Mannheim, Germany). Goat anti-MRP2 polyclonal (Ab) (H-17), rabbit anti-ABCG2 (BCRP) polyclonal Ab (H-70) were purchased from Santa Cruz Biotechnology (Santa Cruz, CA). Mouse monoclonal anti-P-glycoprotein Ab (C219) and mouse monoclonal anti-MVP/LRP Ab (MVP 37) were obtained from Alexis Biochemicals (Lörrach, Germany).

### Cell lines and cell culture

The Human Ovarian Carcinoma Cell Line A2780 was purchased from ATCC. A2780 sublines that were resistant to CIS (A2780CR1, A2780CR2), PAC (A2780PR1, A2780PR2), DOX (A2780DR1, A2780DR2), and TOP (A2780TR1, A2780TR2) were generated by the exposure of the A2780 cell line to incremental increases in the concentrations of the relevant drugs. The final concentrations of each drug were 1000 ng/mL CIS, 1100 ng/mL PAC, 100 ng/mL DOX, and 24 ng/mL TOP. These concentrations were based on the work of Dietel et al. in 1993 [23] and were twofold greater than the plasma concentrations of the respective drugs 2 h after intravenous administration. All the cell lines were maintained as monolayers in complete medium (MEM medium supplemented with 10 % fetal bovine serum, 2 pM L-glutamine, penicillin (100 units/mL), streptomycin (100 units/mL), and amphotericin B (25 μg/mL) at 37 °C in a 5 % CO_2_ atmosphere.

### Drug sensitivity assay

The drug sensitivity of the A2780 cell line and the drug resistant cell lines was confirmed by the MTT cell survival assay. Briefly, all cell lines were seeded at a density of 5000 cells/well in 96-well plates. The cells were allowed to grow for 48 h and subsequently treated with fresh medium supplemented with or without increasing concentrations of drugs and incubated for 72 h at 37 °C. After 72 h of exposure, 10 μL of the MTT labeling reagent was added to the medium (the final concentration of MTT was 0.5 mg/mL), and the cells were incubated for additional 4 h. Following this process, 100 μL of solubilisation solution was added to each well. The absorbance of each sample was measured in a microplate reader at 570 nm with a reference wavelength of 720 nm, according to the manufacturer’s protocol. The negative control was conducted using cell-free culture medium containing both the MTT reagent and solubilisation solution. The experiments were repeated three times, and each concentration in a given experiment was tested in duplicates. Cell viability was expressed as a percentage of the untreated control (means ± SEM).

### Examination of gene expression by using Q-PCR

Changes in *ABCB1*, *ABCC1*, *ABCC2*, *ABCG2* and *LRP* genes expression in the A2780 and drug-resistant cell lines were examined. Q-RT-PCR was conducted for four independent experiments. RNA was isolated using the Gene Matrix Universal RNA purification Kit (EURx Ltd.), as described by the manufacturer. Reverse transcription was performed using the M-MLV reverse transcriptase (Invitrogen) as described in the manufacturer’s protocol using a thermal cycler (Veriti 96 well Thermal Cycler). 2 μg of RNA was used to cDNA synthesis. Real-time PCR was performed using the Applied Biosystems PCR System (7900HT Fast Real-Time PCR System), Maxima SYBR Green/ROX qPCR Master Mix (Fermentas) and sequence-specific primers, as indicated in Table 1. Glyceraldehyde-3-phosphate dehydrogenase (GADPH), β-actin, hypoxanthine-guanine phosphoribosyltransferase 1 (HRPT1) and beta-2-microglobulin (β2M) served as the normalizing genes (geometric mean) against which expression changes in the examined genes were compared. Gene expression was analyzed using the relative quantification (RQ) method. RQ estimates the difference in the level of gene expression against a calibrator (A2780 drug sensitive line) (RQ of the calibrator = 1). The A2780 cell line was used as the calibrator. The analysis was conducted employing the standard formula: RQ = (where for the sample (drug-resistant line) − for the calibrator (drug sensitive line)). The graphs were made using Sigma Plot.

**Table 1:** Oligonucleotide sequences used for Q-PCR analysis

Transcript	Sequence (5’-3’ direction)	ENST number http://www.ensembl.org	Product size (bp)
ABCB1	TGACAGCTACAGCACGGAAG	00000265724	131 bp
	TCTTCACCTCCAGGCTCAGT		
ABCC1	GAGAGTTCCAAGGTGGATGC	00000399410	149 bp
	AGGGCCCAAAGGTCTTGTAT		
ABCC2	TACCAATCCAAGCCTCTACC	00000370449	104 bp
	AGAATAGGGACAGGAACCAG		
ABCG2	TTCGGCTTGCAACAACTATG	00000237612	128 bp
	TCCAGACACACCACGGATAA		
LRP	TGAGGAGGTTCTGGATTTGG	00000357402	135 bp
	TGCACTGTTACCAGCCACTC		
GADPH	GAAGGTGAAGGTCGGAGTCA	00000229239	199 bp
	GACAAGCTTCCCGTTCTCAG		
β-actin	TCTGGCACCACACCTTCTAC	00000331789	169 bp
	GATAGCACAGCCTGGATAGC		
HRPT1	CTGAGGATTTGGAAAGGGTG	00000298556	156 bp
	AATCCAGCAGGTCAGCAAAG		
β2M	CGCTACTCTCTCTTTCTGGC	00000558401	133 bp
	ATGTCGGATGGATGAAACCC		

For amplification, 12.5 μL of Maxima SYBR Green/ROX qPCR Master Mix (Fermentas), 1 μL of each primer (Oligo, Warsaw, Poland) (Table 1), 9.5 μL of water, and 1 μL of cDNA solution were mixed together. One RNA sample of each preparation was processed without RT-reaction to provide a negative control in subsequent PCR. Sample amplification included a hot start (95 °C, 15 min) followed by 50 cycles of denaturation at 95 °C for 15 s, annealing at 60 °C for 30 s, and extension at 72 °C for 30 s. After amplification, Melt Curve analysis was performed to analyze product melting temperature. The amplification products were also resolved by 3 % agarose gel electrophoresis and visualized by ethidium bromide staining.

### Immunofluorescence analysis

The cells were cultured on microscopic glass slides and grown to a near-confluent state. Afterwards, the cells were fixed in 4 % PFA in PBS for 10 min at room temperature, permeabilized in ice-cold acetone/methanol (1:1) for 10 min at −20 °C, rinsed with PBS and blocked in 3 % BSA for 45 min. Several primary antibodies were used for detection including: MRP2 (1:50, 1 h/RT, goat polyclonal anti-human, clone H-17, Santa Cruz Biotechnology), P-gp (1:100, 1 h/RT, mouse monoclonal anti-human, clone C219, Alexis Biochemicals), BCRP (1:100, 1 h/RT, rabbit polyclonal anti-human, clone H-70, Santa Cruz Biotechnology) and MVP/LRP (1:50, 1 h/RT, mouse monoclonal anti-human, clone MVP 37, Alexis Biochemicals) along with the corresponding green dye labeled secondary antibody: anti-goat, anti-rabbit, anti-mouse respectively (MFP488, donkey anti-goat IgG, goat anti-mouse IgG, goat anti-rabbit IgG; 1:200, 1 h/RT, MoBiTec). Afterwards, the cells were washed three times with PBS and sealed with DAPI-containing mounting medium. The cells were viewed under a fluorescence microscope (Zeiss Axio-Imager.Z1). The expression of MRP2, P-gp, BCRP and MVP/LRP was analyzed using pseudo-colour representations of fluorescence intensity for DAPI at 365 nm excitation and 420 nm emission wavelengths (blue) and for MFP488 at 470 nm excitation and 525 nm emission wavelengths (green).

### Flow cytometric analysis

To assess the efflux activity of P-gp, MRP2 and BCRP in drug sensitive and resistant cell lines, we used the fluorescent dye Rhodamine 123 (Rho123) as an index of P-gp and MRP2 activity and Hoechst 33342 (H33342) as an index of BCRP activity. The cell suspensions (1x10^6^/ml) were incubated with 1 μg/ml Rho123 or 1 μg/ml H33342 for 1 h at 37 °C in the medium. Next, the cells were washed twice in ice cold PBS with 500 μM verapamil (a known MDR inhibitor), and cellular uptake of Rho123 or H33342 was immediately analyzed using a FACSAria III (BD, Warsaw, Poland) with the FCS Express Plus software program. In each analysis, 10,000 events were recorded. The fluorescent emission was collected at 488 nm for Rho123 and at 375 nm for H33342.

### Statistical analysis

The statistical analysis was performed using Microsoft Excel software. The statistical significance of the differences was determined by applying the Student's *t*-test.

## Results

### Characteristics of A2780 and A2780 sublines

**Fig. 1:**
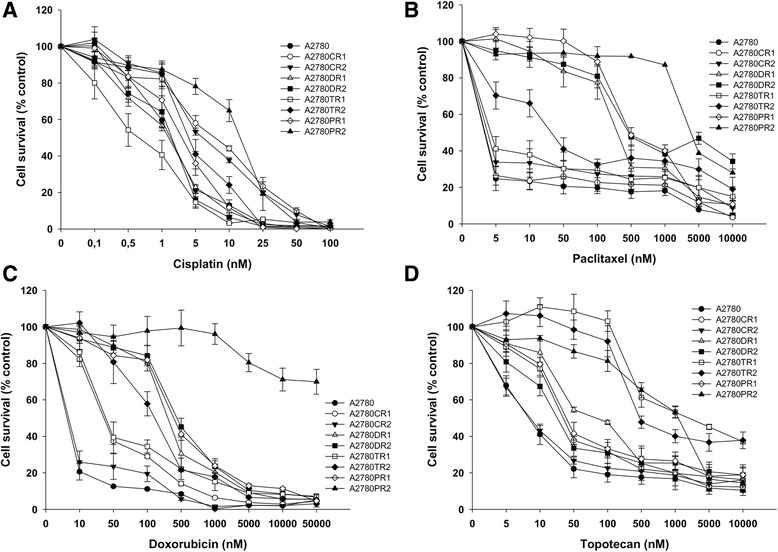
MTT analysis. MTT cell survival assay for A2780, A2780CR1, A2780CR2, A2780DR1, A2780DR2, A2780TR1, A2780TR2, A2780PR1 and A2780PR2 cell lines treated with or without increasing concentration of CIS (**a**), PAC (**b**), DOX (**c**), and TOP (**d**). The viability was expressed as a percent on an untreated control (mean ± SEM)

The CIS-resistant (A2780CR1, A2780CR2), PAC-resistant (A2780PR1, A2780PR2), DOX-resistant (A2780DR1, A2780DR2) and TOP-resistant (A2780TR1, A2780TR2) variant sublines of the A2780 human ovarian cancer cell line were all established by the stepwise selection of A2780 cells cultured in growth media with increasing drug concentrations. To determine the sensitivity of the A2780 and drug-resistant A2780 sublines to CIS, PAC, DOX, and TOP, cells were treated with different concentrations of each drug for 72 h. The concentration- dependent effect of CIS on A2780 and the drug-resistant cell lines was observed (Fig. 1a, Table 2). The A2780DR1, A2780DR2, A2780TR1, A2780TR2 and A2780PR1 cell lines were all sensitive to CIS treatment. In contrast we observed an increased resistance to CIS not only in both CIS-resistant cell lines (A2780CR1 and A2780CR2) but also in A2780PR2 cell line in comparison with parental A2780 cell line.

**Table 2:** Summary of cell line cross-resistance to drug treatment

IC50 (nM)				
Cell line	CIS	PAC	DOX	TOP
A2780	1.83	3.37	6.34	10.8
	(0.94–3.14)	(2.84–3.76)	(5.76–7.04)	(5.76–20.0)
	1	1	1	1
A2780CR1	7,49	3.43	41.5	37.3
	(5.39–8.72)	(3.09–3.93)	(32.8–48.0)	(31.7–43.7)
	4.09 ↑ *	1.01	6.54 ↑ **	3.45
A2780CR2	6.03	3.86	6.84	8.59
	(5.12–7.10)	(3.13–4.48)	(6.07–8.02)	(8.09–9.11)
	3.29 ↑ *	1.15	1.08	0.79
A2780DR1	1.79	283	367	37.3
	(1.64–1.96)	(123–601)	(253–496)	(33.5–41.0)
	0.98	84 ↑ **	58 ↑ **	3.45
A2780DR2	2.32	569	461	30,9
	(0.86–3.06)	(407–983)	(353–596)	(22.1–38.6)
	1.27	169 ↑ **	73 ↑ **	2.86
A2780TR1	0.84	4.13	46.4	644
	(0.36–1.40)	(3.56–4.65)	(34.6–68.6)	(350–851)
	0.46	1.22	7.3 ↑ *	59.6 ↑ **
A2780TR2	4.24	40.5	192	523
	(2.91–4.95)	(24.7–56.6)	(59.4–252)	(409–616)
	2.31	12 ↑ **	30 ↑ *	48.5 ↑ **
A2780PR1	3.41	491	419	61.4
	(1.86–4.57)	(457–534)	(384–464)	(49.9–70.3)
	1.86	146 ↑ **	66 ↑ **	5.68 ↑ *
A2780PR2	15.5	4052	22,673	1398
	(9.31–22.4)	(3872–4452)	(29,757–14,104)	(880–1913)
	8.46 ↑ **	1202 ↑ **	3476 ↑ **	129 ↑ **

IC50 mean is indicated to each drug. The drug resistance in A2780 cell line was assigned as 1. ↑ Underline values indicate multiplicities of resistance with respect to A2780 cell line. *p < 0.01, **p < 0.001

We observed high cross-resistance between PAC- and DOX-resistant cell lines (Fig. 1b and c, Table 2). Also, Both PAC-resistant cell lines (A2780PR1 and A2780PR2) demonstrated very high level of resistance to DOX. Similarly, both DOX-resistant cell lines (A2780DR1 and A2780DR2) were also resistant to PAC. Among other cell lines we observed only medium level of PAC cross-resistance in A2780TR2 cell line (Fig. 1b, Table 2). We also observed low level of cross-resistance to DOX in A2780CR1 and A2780TR1 cell lines and medium level of cross-resistance in A2780TR2 cell line (Fig. 1c, Table 2).

The effect of TOP was also investigated. In A2780TR1 and A2780TR2 we observed high level of TOP resistance (Fig. 1d, Table 2). We also observed cross-resistance to TOP in both PAC-resistant cell lines. Furthermore, A2780PR2 cell lines was more resistant to TOP than A2780TR1 and A2780TR2 cell lines (Fig. 1d, Table 2). Both TOP-resistant cell lines showed cross-resistance to DOX (Fig. 1c, Table 2) and one A2780TR2 cell line showed cross-resistance to PAC (Fig. 1b, Table 2).

### Gene expression analysis in drug-resistant ovarian cancer cell lines

**Fig. 2:**
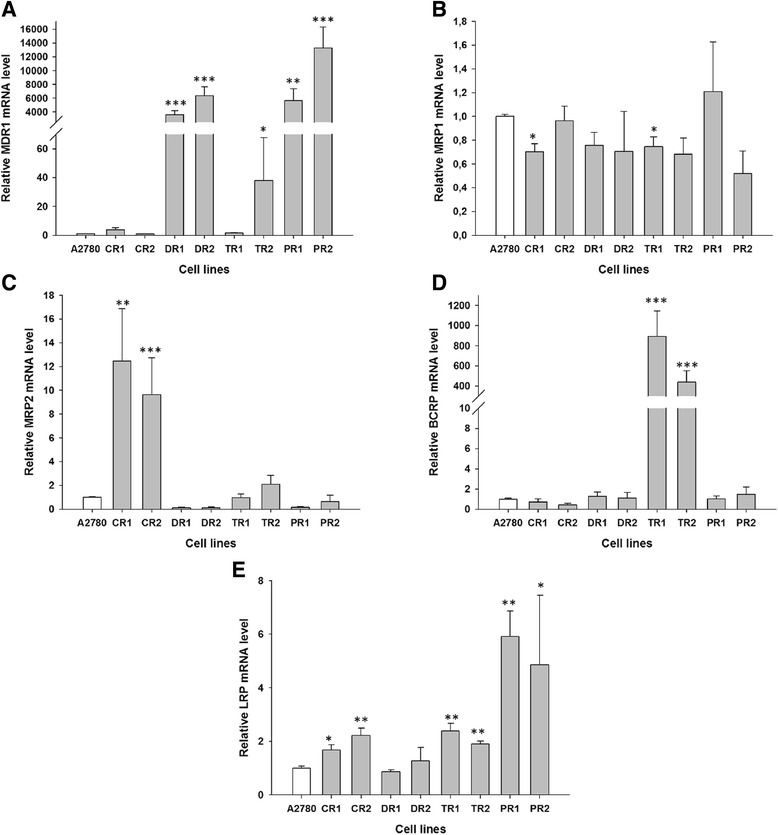
Gene expression analysis. Expression (Q-PCR) of MDR1 (**a**), MRP1 (**b**), MRP2 (**c**), BCRP (**d**), and LRP (**e**) genes. The figure presents relative gene expression in resistant cell lines (*grey bars*) with respect to the A2780 cell line (*white bars*) assigned as 1. Values were considered significant at *P < 0.05, **P < 0.01 and ***P < 0.001

To determine whether the development of drug-resistance in the variant sublines of the A2780 parental line is associated with increased expression of MDR-associated genes, expression of the following mRNA levels was assessed: MDR1, MRP1, MRP2, BCRP, and LRP. We observed statistically significant increase of MDR1 transcript level in both DOX-resistant cell lines (*P* < 0.001), both PAC-resistant cell lines (*P* < 0.01 in A2780PR1 cell line and *P* < 0.001 in A2780PR2 cell line) and one TOP-resistant cell line A2780TR2 (*P* < 0.05) (Fig. 2a). However, expression in A2780TR2 cell line was much lower than in PAC- and DOX-resistant cell lines. The transcript level of MRP1 was significantly decreased in one CIS-resistant cell line (A2780CR1, *P* < 0.05) and one TOP-resistant cell line (A2780TR1, *P* < 0.5) (Fig. 2b), however, these changes were very low. MRP2 expression increased in both CIS-resistant cell lines (*P* < 0.01 in A2780CR1 cell line and *P* < 0.001 in A2780CR2 cell line) (Fig. 2c). Both TOP-resistant cell lines were characterized by significantly increased BCRP transcript level (*P* < 0.001) (Fig. 2d). The LRP transcript level was significantly higher in both CIS-resistant cell lines A2780CR1, and A2780CR2 (*P* < 0.05 and *P* < 0.01, respectively), both TOP-resistant cell lines (*P* < 0.01) and both PAC-resistant cell lines A2780PR1 and A2780PR2 (*P* < 0.01 and *P* < 0.05, respectively) (Fig. 2e), however the increase was not very high.

### Immunofluorescence of MRP2, P-gp, BCRP and LRP in resistant cell lines

**Fig. 3:**
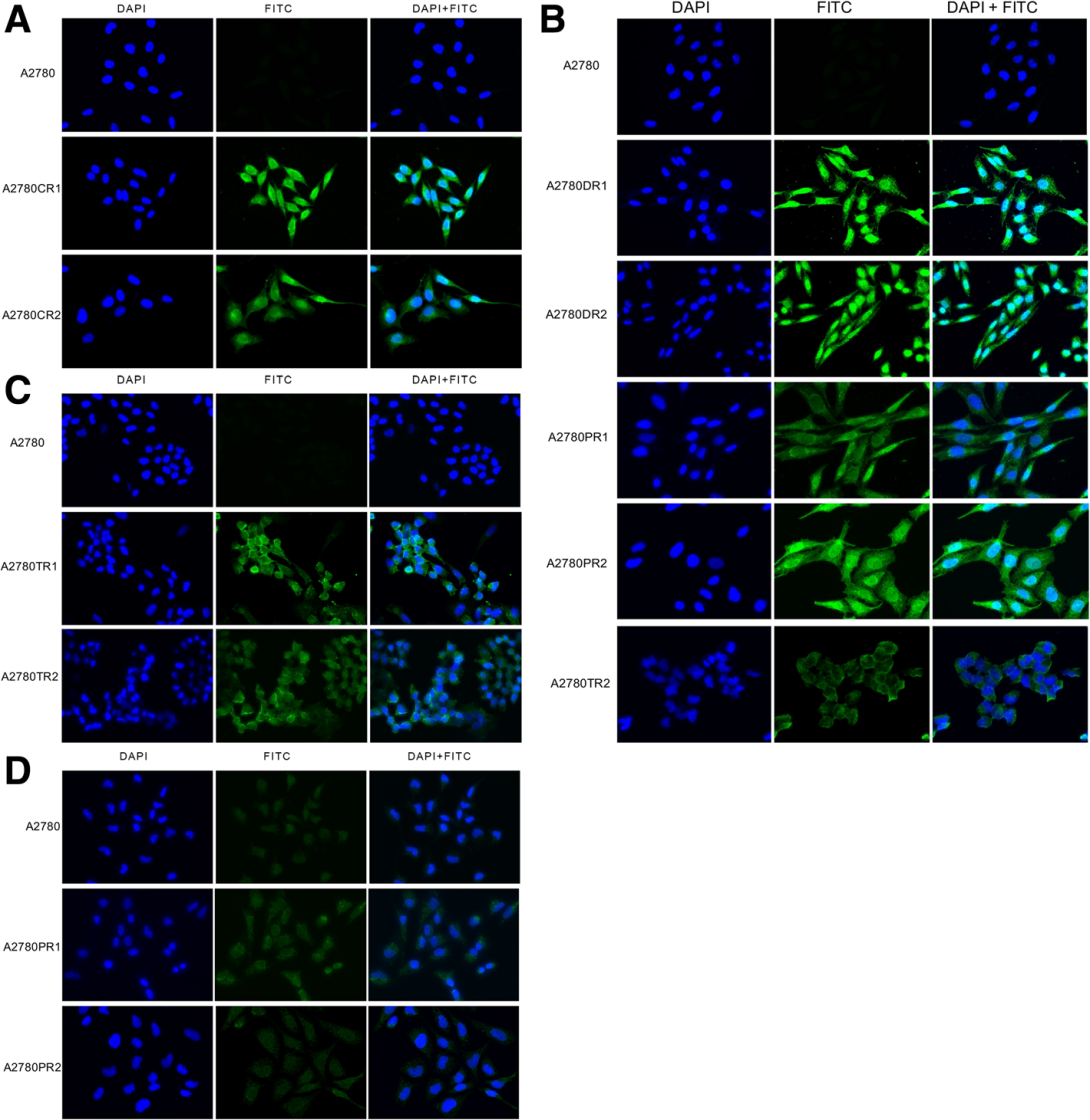
Immunofluorescence analysis. Immunofluorescence visualization of: (**a**) MRP2 protein in A2780, A2780CR1 and A2780CR2 cell lines; (**b**) P-gp protein in A2780, A2780DR1, A2780DR2, A2780PR1, A2780PR2 and A2780TR2 cell lines; (**c**) BCRP protein in A2780, A2780TR1 and A2780TR2 cell lines; (**d**) LRP/MVP protein in A2780, A2780PR1 and A2780PR2 cell lines. All antigens were detected using respected primary antibodies and corresponding MFP488-conjugated secondary antibodies (*green*). To visualize the cell nuclei, the cells were mounted with a DAPI-containing mounting medium (*blue*). Objective x40

To confirm the expression of investigated drug transporters at the protein level, we used the immunofluorescence assay. The immunofluorescence analysis validated the transcript expression results. We observed an increased expression of MRP2 protein in the A2780 sublines resistant to CIS (Fig. 3a). Similarly, the expression of P-gp protein was observed in cell lines resistant to DOX (A2780DR1 and A2780DR2) and PAC (A2780PR1 and A2780PR2) and at lower level in A2780TR2 cell line resistant to TOP (Fig. 3b). We also observed expression of BCRP protein in both TOP resistant cell lines (A2780TR1 and A2780TR2) (Fig. 3c). The increase in MVP transcript level in CIS- and TOP-resistant cell lines was very low, therefore we compared the protein expression only between A2780 and both PAC-resistant cell lines, where we could observe low increase in expression of MVP protein (Fig. 3d).

### Analysis of drug transporters activity in drug resistant cell lines

To determine whether expression of MRP2, P-gp and BCRP correlates with their transporter activity or not the fluorescence accumulation was investigated in drug sensitive and resistant cell lines. The MRP2 and P-gp activity was examined with the use of day Rho123 and activity of BCRP with the use of day H33342, respectively.

**Fig. 4:**
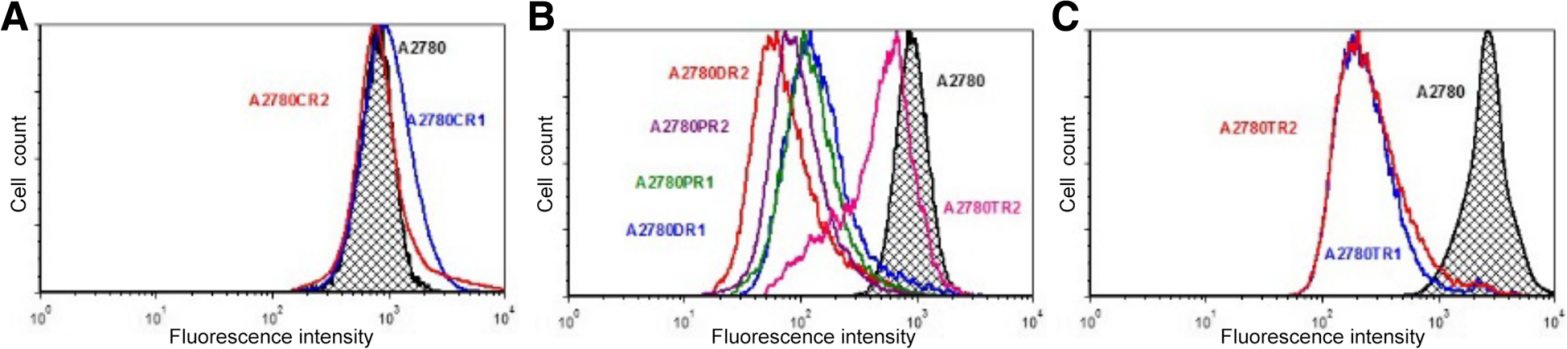
Intracellular accumulation of Rho123 and H33342 in drug sensitive and resistant cell lines. The figure shows diagrams of the fluorescence intensity, each consisting of the fluorescence intensity of the sensitive cell line and corresponding resistant cell lines. Sensitive cell lines are indicated in black. Accumulation of Rho123 in cell lines with expression of MRP2 (**a**) and in cell lines with expression of P-gp (**b**). Accumulation of H33342 in cell lines with expression of BCRP (**c**)

**Fig. 5:**
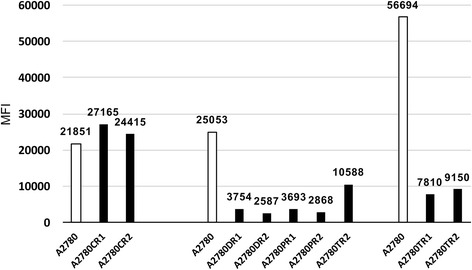
MRP2, P-gp and BCRP activity analysis. Mean fluorescence intensity (MIF) of Rho123 in A2780, A2780CR1 and A2780CR2 cells for MRP2 activity and in A2780DR1, A2780DR2, A2780PR1, A2780PR2 and A2780TR2 cells for P-gp activity. Mean fluorescence (MIF) of H33342 in A2780, A2780TR1 and A2780TR2 cells for BCRP activity. The MFI was calculated automatically using the FACS Express Plus software program

We did not observe increased activity of MRP2 in A780CR1 and A2780CR2 cell lines in comparison with drug sensitive A2780 cell lines (Fig. 4a). Furthermore, the accumulation of Rho123 was slightly higher in both resistant cell lines (Fig. 5). In cell lines resistant to PAC and DOX we observed much lower level of Rho123 accumulation than in drug sensitive cell line A2780 (Fig. 4b). A2780TR2 cell line was also characterized by lower level of Rho123 accumulation than A2780 cell lines, however, the differences were much lower than in PAC- and DOX-resistant cell lines. The accumulation of Rho123 in DOX-resistant cell lines A2780DR1 and A2780DR2 was respectively 6.7 and 9.7 fold lower than in drug sensitive A2780 cell line (Fig. 5). Similarly, the accumulation in PAC-resistant cell lines A2780PR1 and A2780PR2 was respectively 6.8 and 8.7 fold lower than in drug sensitive A2780 cell line (Fig. 5). In A2780TR2 cell line we observed only 2.4 fold lower accumulation of Rho123 than in A2780 cell line. In both TOP-resistant cell lines (A2780TR1 and A2780TR2) we observed decreased accumulation of H33342 (Fig. 4c). The accumulation of H33342 in A2780TR1 and A2780TR2 cell lines was respectively 7.3 and 6.2 fold lower than in drug sensitive A2780 cell line (Fig. 5).

## Discussion

In the present study we investigated the development of resistance to cytotoxic drugs after exposure the ovarian cancer cell line A2780 to cytotoxic drugs used in the treatment of this cancer. The most important cytotoxic drug commonly used in ovarian cancer treatment is CIS [18, 19]. It was previously described by others that ovarian cancer cells can develop a special metabolic mechanisms of resistance to cisplatin [24], however simultaneous cross-resistance to another drug was not observed in those cell lines. We could observe increased resistance to CIS not only in both CIS-resistant cell lines but also in A2780PR2 cell line resistant to PAC. PAC is the second most important drug in the first line chemotherapy of ovarian cancer [19, 20]. Cross-resistance of PAC-resistant cell line to CIS can suggest that in patients who developed resistance to PAC, neither CIS can be an effective drug in cancer treatment.

High level of cross-resistance between PAC- and DOX-resistant cell lines is not surprising because cross-resistance between cancer cells resistant to these drugs has been documented by others [3, 6] and ours [25] previously. High level of cross-resistance between cell lines resistant to these drugs suggests that DOX based chemotherapy should not be recommended for patients that developed resistance to PAC after the first line chemotherapy.

Another drug that is commonly used in many cancers, including second line chemotherapy in ovarian cancer, is topotecan [18, 21]. Similar pattern of response to TOP was observed in both TOP-resistant cell lines, and in A2780PR2 cell line. A2780PR1 cell line also showed TOP resistance, although at much lower level. High level of resistance to TOP in PAC resistant cell line was previously observed by ours in another ovarian cancer cell lines study [25]. Cross-resistance of PAC-resistant cell lines to TOP raises the question whether TOP is a proper drug for a second line of ovarian cancer chemotherapy or not.

The most important MDR protein is P-gp encoded by *MDR1* gene [4]. We observed very high level of MDR1 transcript in both PAC- and both DOX- resistant cell lines. In A2780TR2 cell line MDR1 transcript level was also increased in comparison with the control cell line, however, at much lower level. Very similar results were obtained at protein level. Drug transporter activity of P-gp determined by Rho 123 efflux was also higher in both PAC- and DOX- resistant cell lines and in A2780TR2 cell line than in control. Increased expression and activity of P-gp in DOX- and PAC- resistant cell lines is not surprising because both drugs are well known substrates for P-gp [3, 4, 7, 8, 26]. Similarly to the results of our previous studies [25] in current research we could observe very high correlation between MDR1 transcript level, P-gp activity and IC50 in DOX- and PAC- resistant cell lines. These results confirm that P-gp plays most important role in the resistance to both cytotoxic agents. Cross-resistance of A2780TR2 cell line to DOX and PAC can also result from P-gp overexpression in this cell line.

It is worth mentioning, that we also observed increased MRP2 transcript level and protein expression in both CIS-resistant cell lines. Expression of MRP2 in CIS-resistant cell lines was also observed by others [27, 28]. Additionally, another cell line – A2780PR2 – showed resistance to CIS, however, statistically significant increase in MRP2 transcript level was not observed in that cell line. In contrast to P-gp, that is considered as a main player in PAC and DOX resistance, the MRP2 seems not to be the one and only important mechanism in CIS resistance. It has been reported that metallothioneins [29], glutathione [30] and glutathione metabolizing enzymes [31], are also responsible for resistance to this drug. Thus, cross-resistance of A2780PR2 cell line can result from one of those mechanisms. Although MRP2 transcript and protein expression were upregulated in both CIS-resistant cell lines, the Rho123 accumulation was higher in both CIS-resistant cell lines than in drug sensitive A2780 cell line. This can result from the fact that Rho123 can also be used as a substrate for other proteins from ABC transporters family. Previously, we have reported downregulation of ABCA3 in both CIS-resistant cell lines [32]. Thus, together with the upregulation of MRP2, the downregulation of ABCA3 in these cell lines occurs and can result in impaired transport and elevated accumulation of Rho123 in CIS-resistant cell lines in comparison with control.

The role of BCRP in resistance to TOP seems to be well established [11, 33] and is confirmed by our results in the present and the previous study [25]. Relation between fluorescence intensity and transcript levels observed in our experiment suggest that BCRP plays an important and a leading role in TOP resistance. However, we observed that both TOP-resistant cell lines were also cross-resistant to DOX. As mentioned previously, the resistance of A2780TR2 cell line to DOX and PAC can be related to P-gp expression, but in contrast to A2780TR2 increased expression of P-gp in A2780TR1 cell line was not observed. Thus, it can be concluded that resistance to DOX in A2780TR1 cell line is related to BCRP expression. That kind of DOX resistance has been reported previously by others and is consistent with data that DOX but not PAC is a substrate for BCRP [4, 6].

Among all our resistant cell lines we could observe two different mechanisms of TOP resistance. Both TOP-resistant cell lines showed “classical” mechanism of TOP-resistance based on BCRP expression. It appears in opposition to both PAC-resistant cell lines where resistance to TOP seems to be related to P-gp expression. It has been reported that TOP is a substrate for P-gp [6] and expression of P-gp can protect cells against TOP [25, 34]. However, both DOX-resistant cell lines also showed very high level of P-gp expression but were not resistant to TOP. Similar observation was made by ours previously in another DOX- and VIN- (vincristin) resistant ovarian cancer cell lines. Although both cell lines expressed high level of P-gp they were not resistant to TOP [25]. This suggests that expression of P-gp can be important but not sufficient for TOP resistance. P-gp mechanism of TOP resistance requires further investigation.

Another question is about the reason of low level of cross-resistance to DOX in A2780CR1 cell line and total lack of DOX-resistance in A2780CR2 cell line. In A2780CR1 cell line we could not observe any positive expression of P-gp or BCRP. It has been previously reported that MRP2 can also be related to DOX resistance [6]. In our experiment the expression of MRP2 in A2780CR1 cell line was slightly higher than in A2780CR2 cell line and can be the reason of low level of resistance to DOX in this cell line.

Another protein that is involved in MDR but does not belong to ABC drug transporters family is LRP/MVP [15]. In our experiment we have observed that LRP transcript level has risen in all examined CIS-, PAC- and TOP-resistant cell lines, but not in DOX-resistant cell lines. Increased expression in CIS-resistant cell lines is consistent with data of Berger et al., who observed a correlation between LRP expression and resistance to CIS in NSCLC cell lines [17]. However, in contrast to other study [35], LRP is evidently not involved in DOX-resistance in our cell lines. Among all examined cell lines the highest expression of LRP was observed in PAC-resistant cell lines. The role of that protein in resistance to PAC has been described by Tegze et al., who observed a correlation between LRP expression and resistance to PAC in breast cancer cell lines [36]. To our knowledge, the role of LRP in TOP resistance has not been described by others so far. Since the increase in LRP expression in drug resistant cell lines was significantly lower than the expression of ABC drug transporters it can appear that LRP plays more of a complementary and not the main role in MDR. This is consistent with results of SiVa et al., who concluded that upregulation of LRP alone is not sufficient to influence the drug resistance phenotype [37].

All drug resistant cell lines were developed from the same A2780 drug sensitive cell line and, what is more, all twin cell lines were resistant to the same cytotoxic drug but we could still observe some particular differences. From two PAC-resistant cell line only one - A2780PR2 - was cross-resistant to CIS and, similarly, from both TOP-resistant cell lines only A2780TR2 revealed increased level of P-gp and resistance to PAC.

Well-described standard response of cancer cells to drugs results in increased expression of typical drug resistance proteins. However, on the basis of our results we can conclude that cancer cells are able to develop an alternative and less specific pathways of response to drug induced stress that leads to cytotoxic drugs cross-resistance. The phenomenon of cross-resistance of cancer cells that has developed effective mechanisms against different types of cytotoxic drugs can directly influence the effectiveness of chemotherapy in ovarian cancer patients.

## Conclusions

In summary, our results confirm that expression of drug transporters from ABC family is the main mechanism of MDR in cancer cells. It is possible to predict drug cross-resistance when the classical mechanism of MDR based on P-gp expression is involved. We observed two mechanisms of TOP resistance: classical - based on BCRP expression in TOP-resistant cell lines and non classical - related to P-gp expression in PAC-resistant cell lines. Effectives of TOP and DOX in the second line of chemotherapy in ovarian cancer can be limited because of their cross-resistance to PAC. Moreover, LRP/MVP seems to play a complementary role in resistance to cytotoxic drugs. Cross-resistance of PAC-resistant cell line to CIS suggests that such cross-resistance between those drugs is also probable in clinic. Although the main mechanisms of resistance in examined twin cell lines resistant to the same cytostatic were similar, we could still observe some differences between them.
